# Sensory Environments for Behavioral Health in Dementia: Diffusion of an Environmental Innovation at the Veterans Health Administration

**DOI:** 10.1177/1937586720922852

**Published:** 2020-06-18

**Authors:** Lesa Lorusso, Nam-Kyu Park, Sheila Bosch, I. Magaly Fretes, Ronald Shorr, Maureen Conroy, Sherry Ahrentzen

**Affiliations:** 1 Gresham Smith, Nashville, TN, USA; 2 College of Design, Construction and Planning, University of Florida, Gainesville, FL, USA; 3 Center of Innovation on Disability and Rehabilitation Research, U.S. Veterans Health System, USA; 4 Geriatric Research Education and Clinical Center, U.S. Veterans Health Administration, USA; 5 University of Florida, Gainesville, FL, USA

**Keywords:** dementia, multisensory, sensory, interview, innovation

## Abstract

**Objectives:**

To evaluate the diffusion of multisensory environments (MSEs) as an innovation at the Veterans Health Administration (VHA) and gather feedback regarding staff perceptions of barriers to uptake and effectiveness of MSEs for Veterans with dementia.

**Background:**

Responding to the need for nonpharmacological behavioral interventions, VHA funded the first MSE for Veterans with dementia in 2010. The room incorporated LED color-changing lights, bubble tubes, vibroacoustic furniture, music, and aromatherapy, and the success of this patient-centered sensory room fueled national rollouts in 2013 and 2015.

**Method:**

A qualitative interview approach was used. Thirty-two staff members participated from 12 of the 53 sites producing 21 individual interviews and 1 group interview with 11 participants. Results were analyzed by a team of eight researchers using the rapid qualitative inquiry method to identify common themes and major insights. Results: Important insights emerged with regard to staff members’ perceptions about the effectiveness of MSE therapy as well as barriers to uptake and suggested strategies for overcoming those barriers (e.g., empowering a champion, developing a clear maintenance plan).

**Conclusions:**

The findings from this research indicate MSEs are perceived as effective in improving behavior for Veterans with dementia and represent an innovation that has been well-diffused within the VHA, with great potential for future clinical applications.

## Background

People living with dementia often have difficulty adapting to environmental stress which negatively impacts their behavioral health and manifests in challenging behaviors causing significant burden to caregivers (Cohen-Mansfield & Parpura-Gill, 2007; Kovach, 2000). These behaviors are often exacerbated for Veterans who have a 30% lifetime prevalence of post-traumatic stress disorder (PTSD) and are therefore twice aslikely to develop dementia (Pinciotti et al., 2017). The connection between PTSD and dementia is thought to be combat trauma, traumatic brain injury, and substance abuse (Qureshi et al., 2010). Veterans with both PTSD and dementia have increased risk of neuropsychiatric behavioral disturbances including sleep disorders, irregular mood, anger, and aggression (American Psychiatric Association, 2013). This poses a critical concern for large healthcare systems like the Veterans Health Administration (VHA) which estimates the number of U.S. Veterans with dementia to reach 761,603 by 2029 (U.S. Deputy Under Secretary for Health for Policy and Planning, 2013). Due to the high cost and dangerous side effects of drug-based interventions, nonpharmacological solutions for behavioral health in dementia are much needed (Nielsen et al., 2017), and environments that address sensory preferences may significantly reduce challenging behaviors (Kovach, 2000). Evidence-based design research indicates the built environment plays an integral role in elevating quality of life for people with dementia through furniture design, spatial layout, and lighting (Ahrentzen & Tural, 2015; Bosch & Gharaveis, 2017), which supports multisensory environments (MSEs) as nonpharmacological behavioral interventions.

MSEs are specially designed environments that use customizable lighting, furniture, and equipment to calm users by addressing multisensory preferences (Jamshidi Manesh et al., 2015; Lorusso & Bosch, 2017). Common elements within these environments include ergonomic vibroacoustic furniture, glass tubes filled with water and bubbles that change color, and LED lighting displaying a variety of colors, music, fiber optics, and aromatherapy. Initial findings indicate MSEs positively impact behavior for people with dementia (Collier et al., 2010; Yao & Algase, 2006), but research is sparse and applications are often so varied in the literature that it is difficult to draw practical conclusions for clinical use (Lorusso & Bosch, 2017).

Thanks to an ongoing commitment to innovation in dementia care, the VHA has provided a unique opportunity to investigate MSEs as behavioral dementia therapy. In 2013, the VHA provided MSE equipment in either fixed rooms or mobile cart applications that was purchased from a common vendor. The organization also provided dementia training to over 53 sites. This offered an unprecedented opportunity to evaluate many comparable MSEs within a single organization. The purpose of this qualitative study is to understand staff perceptions of barriers to uptake of MSEs and effectiveness of MSEs at the VHA for Veterans with dementia.

### Research Purpose

The specific aims of this investigation were to:

1. explore staff perceptions of the effectiveness of MSE as a therapy for Veterans with dementia; and
2. identify barriers to implementing MSE therapy and strategies for overcoming them.

### Diffusion of Innovation (DOI)

**Figure 1.**
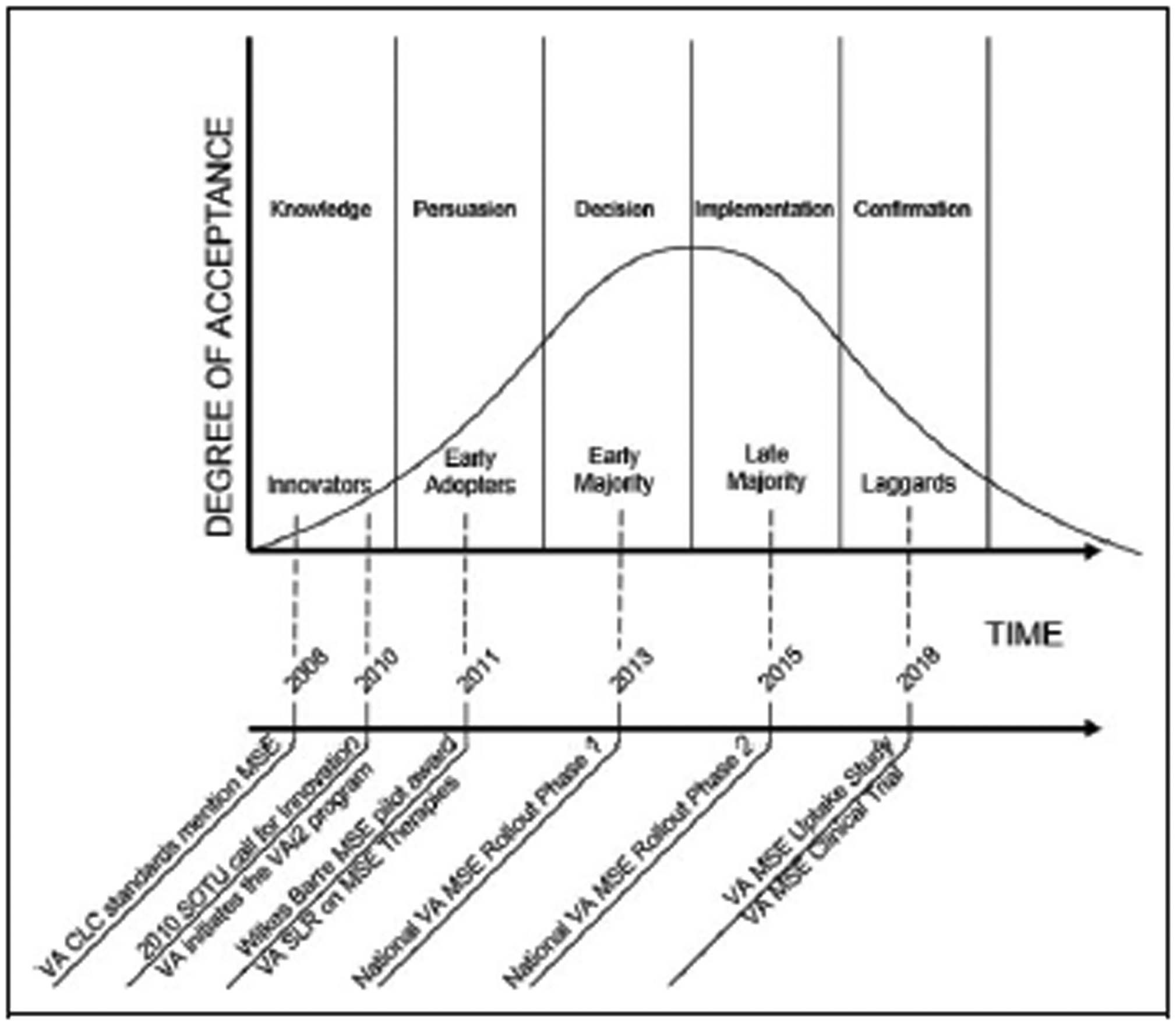
Rogers (2010) diffusion of innovation theory applied to the process of uptake of multisensory environment at the Veterans Administration.

We examined the rollout of MSE equipment in the VHA through the lens of Roger’s (2010) DOI theory, which measures the rate of successful adoption of a new concept, technology, or idea throughout an organization or social structure. Figure 1 illustrates how the VHA’s adoption of MSE for dementia care has progressed through the DOI phases of knowledge, persuasion, decision, implementation, and confirmation.

## Knowledge

According to the DOI theory, the concept of knowledge as a step in the diffusion process refers to when an organization becomes aware of an innovation and develops an initial understanding of what it is and how it functions. This occurred in 2008 when the VHA mentioned MSE within the Criteria and Standards Handbook for VHA Community Living Centers (CLC; VA, 2008). Within the handbook, alternative behavior management strategies were discussed under the topic of considerations for care. MSEs were mentioned since they could “provide alternative behavior management strategies; massage, aromatherapy, music, visualization, and Snoezelen therapy are examples of proven strategies” (Veterans Administration, 2008). Spanning the steps of knowledge and persuasion, the VHA created the Veterans Administration innovation initiative in 2010 following President Obama’s call for innovation during his State of the Union address (Obama, 2010; Veterans Administration, 2012). This laid the foundation for the next phase in the MSE diffusion process.

### Persuasion.

According to DOI theory, persuasion occurs after the knowledge phase when an individual or organization develops a positive or negative opinion regarding an innovation. In 2011, a recreational therapist won a VHA grant to implement MSE therapy and training for Veterans with dementia (Boyle, 2012). This financial support of a pilot program utilizing MSE for Veterans with dementia is evidence of the VHA developing a positive opinion regarding MSE as an innovation and the organization’s commitment to implementing such innovations within dementia care. The Wilkes Barre, PA, CLC successfully persuaded the VHA to make an investment in an early implementation strategy of the innovation, becoming an “early adopter” of the MSE innovation.

### Decision.

The next step in the DOI process is referred to as “decision.” During this phase, the organization actively engaged in activities toward either adopting or rejecting the innovation. Following the success of the pilot MSE program at the Wilkes Barre, PA, CLC, the VHA demonstrated a decision to adopt the MSE innovation by instituting the first phase of a national rollout of MSE for Veterans with dementia in 2013 (Thomas Lynch, email communication, April 23, 2013). This call for participants attracted nearly 53 early majority adopters, following the initial pilot program in Wilkes Barre, PA.

### Implementation.

The two steps of the national MSE rollout spanned the decision and implementation phases of the DOI process. The implementation phase occurs when an organization puts an innovation to use and represents a time when reinvention of the design of innovation is likely to occur. This occurred in 2015 when the VHA sent out a second call for interest, referred to as “National VHA MSE Rollout Phase 2,” as shown in Figure 1. The goal of this second call was to seek additional CLC facilities interested in the MSE program, and it was offered on a first-come, first-served basis through January 23, 2015 (Lisa Minor, email communication, January 15, 2015). This supports the VHA’s continuing commitment to the diffusion of the MSE innovation on a national scale.

### Confirmation.

The confirmation stage occurs when an organization seeks reinforcement of an innovation decision previously made. In this phase, the organization seeks an evaluation of the innovation to understand its effectiveness and reduce uncertainty regarding the innovation’s expected success or results. This investigation is a component of the confirmation phase for the MSE innovation.

## Research Design and Methods

### Sample and Setting

After approval was granted for the study from both the Veterans Administration health services research and development service and the institutional review board (IRB), participants were recruited by a flyer emailed to key medical personnel at each of 53 CLC across the United States and Puerto Rico. The assistant deputy under secretary of health for clinical operations and VHA leadership sent the flyers to the potential participants. CLC are the VHA equivalent of assisted living facilities. CLC are healthcare facilities where Veterans with dementia live and where the MSEs were provided. Forty-one participants responded from 12 of 53 sites; 9 participants were removed due to inclusion/exclusion criteria. A final total of 32 VHA staff from the 12 response sites participated in this study comprising 21 individual interviews and 1 group interview with 11 participants. According to IRB requirements, every participant was assigned a number during the de-identification process explanation of participation rates is discussed within the limitations section. Table 1 provides a list of study sample characteristics including response rate, settings, and number of responses per site. Further explanation of sample characteristics.

### Instrument

An interview guide was sent to participants prior to the interview, so the interviewee had time to reflect upon possible responses. The guide consisted of four sections including a total of 32 questions. Section 1 consisted of 12 multiple-choice questions designed to gather operational data on the CLC and the participant’s level of involvement with the MSE including the type of facility, the participant’s length of experience at the facility, date the MSE was received, and what specific MSE was received. Section 2 consisted of 13 multiple-choice questions designed to gather clinical data regarding the use of MSEs at each CLC such as information on responsible persons using the MSE, whether training was provided to staff, and information on implementation and documentation methods. Section 3 consisted of six open-ended, fill-in-the blank, and Likert-type items intended to ascertain barriers to MSE uptake, Veteran-preferred MSEs and staff perceptions of the overall effectiveness and characteristics of the MSE. Section 4 consisted of one open-ended question designed to allow the participant to provide open-ended comments regarding their own lessons learned and suggestions for other facilities interested in utilizing MSEs for people with dementia. The interview guide incorporated feedback from upper administration from the VHA to reduce researcher bias and ensure validity and reliability (Creswell, 2017) and, as an initial pilot test, was sent to five VHA research staff to test for completion time and appropriateness of content.

### Procedure

Interested participants contacted the research team directly and were provided with the approved waiver of documentation of informed consent. The interview guide was provided via email prior to the interview along with the informed consent form waiver and a brief description of the study. In doing so, the participants had the opportunity to complete the interview guide before the interview and thoughtfully develop a response for the open-ended discussion. The research team consisted of eight members, and the primary researcher conducted the telephone interviews between June and August 2018. The interviews took approximately 20–30 min to complete, were conducted over the telephone, and were recorded on digital audio recording devices encrypted with passwords. The audio files were stored behind the VHA’s firewall, and participants were de-identified using a cross-walk which provided each participant with a specific number. The interviews were transcribed verbatim into word processing files for analysis and labeled by participant number.

### Data Analysis

Interview transcripts were analyzed by a team of eight researchers following the rapid qualitative inquiry (RQI) method (Beebee, 2014). The team consisted of one primary, three secondary, and four tertiary members based on responsibility during the coding process. According to Beebee (2014), the RQI method is an intensive, team-focused qualitative method that aims to understand an insider’s perspective on a topic using multiple data sources and triangulation with an iterative data analysis process to develop an understanding of a topic within a relatively short amount of time. The term “rapid” does not mean rushed but rather refers to the fact that the method allowed a team of at least two to analyze data in a focused way within 6 weeks. Once the recorded interviews were transcribed verbatim by the primary member, the de-identified transcripts were provided to the secondary team members for review.

**Figure 2.**
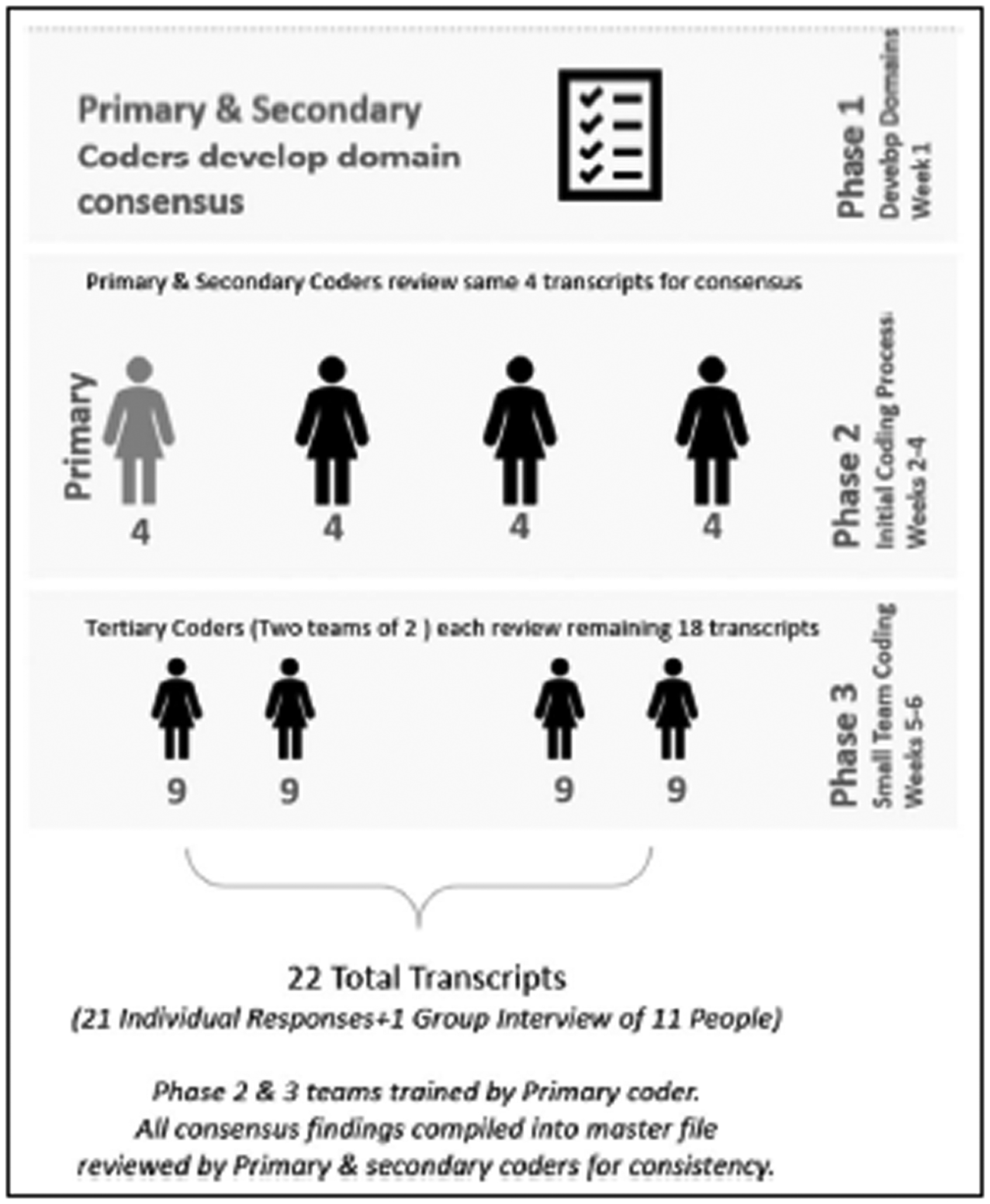
Coding analysis process diagram.

The team developed domains based on the content of the interview guide organized into areas including equipment, treatment, training, barriers, MSE preference of Veteran with dementia, effectiveness of MSE, staff satisfaction, and overall lessons learned. The analysis process is illustrated in Figure 2. Any disagreements were discussed until consensus was agreed upon by the team, and results were analyzed for emerging themes. Section 3 of the interview guide consisted of questions intended to learn staff’s perceptions of Veteran’s preferences of MSE equipment and effectiveness of the MSE. Responses from Section 3, questions two, and three were entered into excel for analysis.

## Findings

Key findings have been organized according to our specific research aims. In an effort to clarify the weight of the narrative responses provided, three descriptive categories were used: few (<9), several (9–17), and many (>17).

### Exploring Staff Perceptions of MSE Effectiveness (Aim 1)

#### Positive effects of MSE.

Although few participants experienced negative effects from the MSE, all 32 participants saw some type of positive effect. Many noted positive distraction, several mentioned engagement, and a few noted marked behavior improvement in the form of reduced agitation, disruptive behavior, and aggression. One respondent commented:

I’ve been able to see significant behavior changes … you can take somebody who’s just completely agitated, confused, irritable or who’s having sun-downing or adjustment issues … and then by bringing them into a sensory environment and engrossing them in the environment will help relax them. (Participant 25, Recreation Therapist)

Another staff member highlighted the effective MSE application to a variety of behaviors by saying: “We use it for redirecting. The MSE we have is a great asset to our facility. It has brought disruptive behavior down and we continue to use it every day” (Participant 28, Nursing Assistant). Some respondents cited the positive impact of MSE for problem behaviors other than aggression such as an overall decreased interest in or lack of engagement with the world. One staff member noted the MSE’s positive impact on engagement by saying: “It gives us an extra edge as to what we can provide Veterans who tend to be nonverbal” (Group Session 1).

Music and aromatherapy also seemed too many staff members as an important MSE element that impacted engagement through reminiscence. One respondent mentioned that:

The dementia patients may not be able to talk … but if you put music on that was around their time you would be surprised that they knew the words … they remember the smells from childhood. It’s really nice. They might say, “Oh, that smells like the cinnamon rolls my mom made.” (Participant 31, Nursing Assistant)

Many of the staff respondents felt that a primary benefit to the MSE was its overall calming effect on Veterans with dementia. Common elements often described as contributing to a calming effect included music, the bubble tubes, and aromatherapy. Participants regularly commented that the MSE caught their attention, eased their minds, and promoted reminiscence. Bubble tubes were often cited as an element within the MSE that seemed to contribute directly to the feeling of calm and a reduction of anxiety levels. Some respondents also commented that the MSE had a positive impact on physical engagement and positive distraction. One respondent stated, “You can dim the lights and play music which is relaxing, and it kind of takes the [patient’s] focus off the pain and he focuses on the music” (Participant 4, Recreational Therapist).

**Figure 3.**
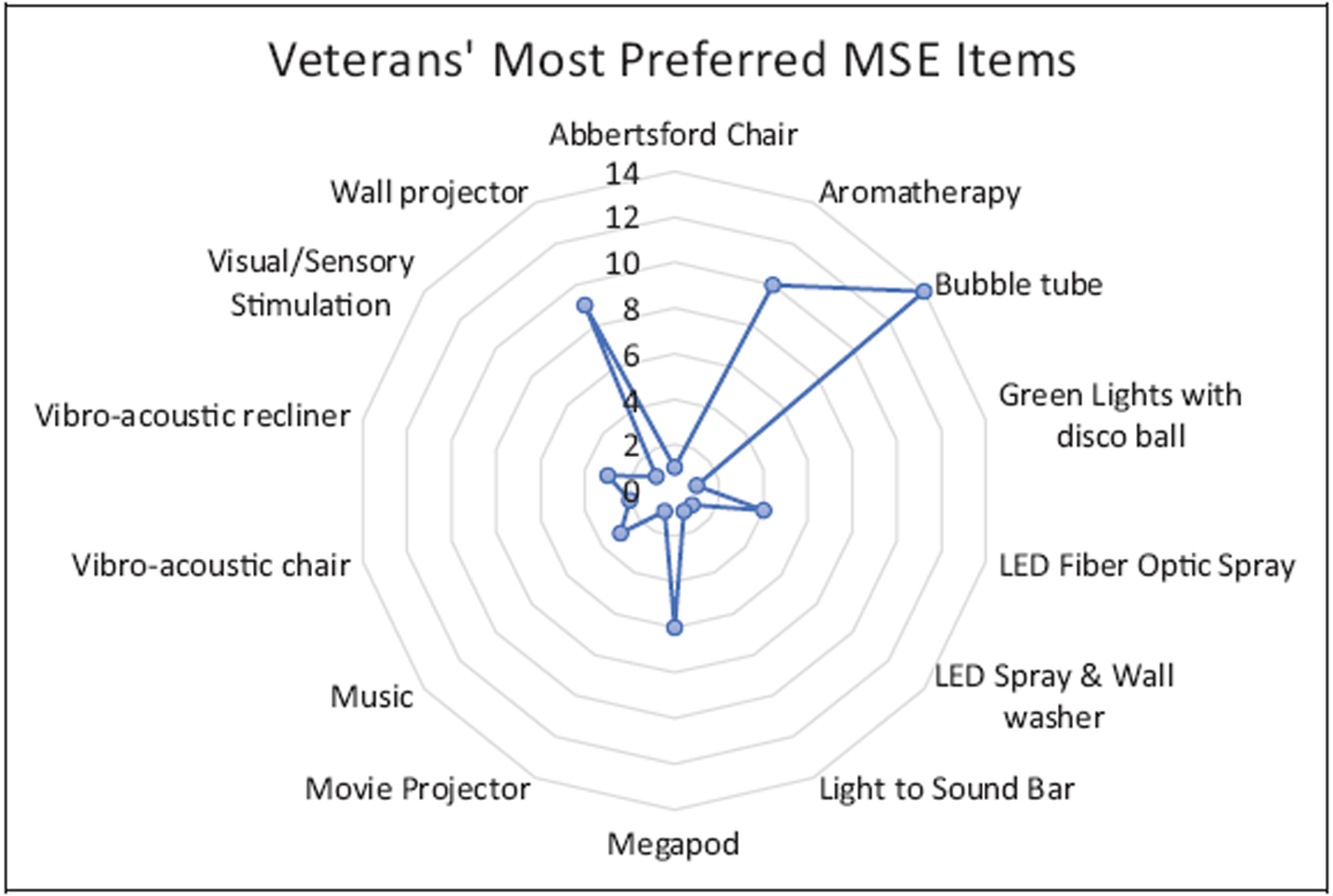
Veterans’ most preferred sensory items within the multisensory environment, from the perspective of the Veterans Administration staff.

Participants were asked to rate the MSE items that they believed the Veteran preferred and then rate in their own opinion as to which MSE equipment they felt were most therapeutically effective for the Veteran at their facility to see whether there were any major differences or overlaps. As seen in Figure 3, the three most preferred sensory items within the environments were the bubble tubes, aromatherapy, and the wall projector, followed by the Megapod, LED fiber optic spray, vibroacoustic furniture, and music. This information is based on participants’ self-reported observations of the equipment within the MSE that Veteran either request the most or seem to gravitate toward.

**Figure 4.**
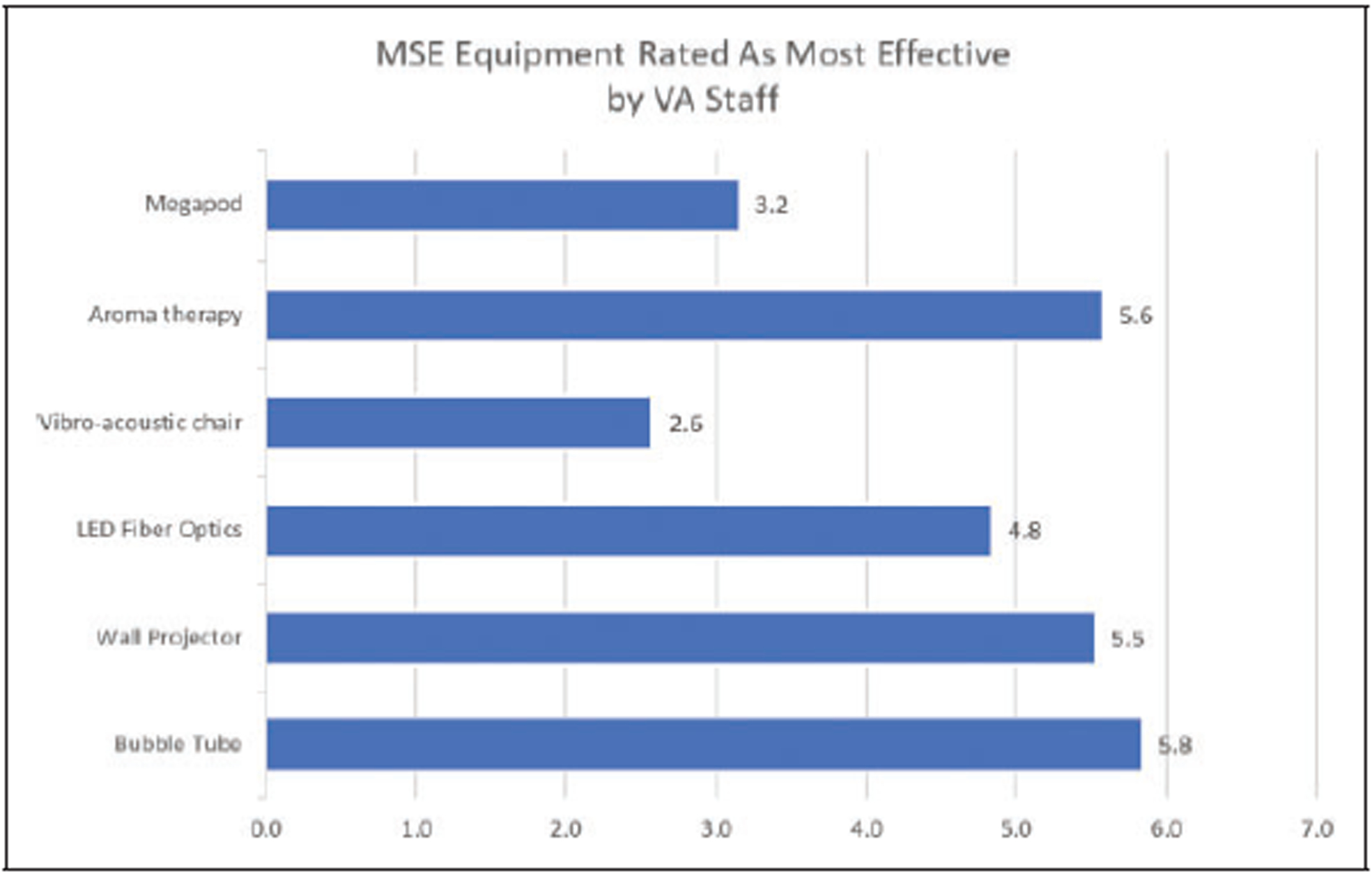
Veterans Administration staff rating of the effectiveness of multisensory environment equipment.

Participants were also asked to rate their own preference regarding which MSE elements were the most therapeutically effective for the Veterans. Staff rated the aromatherapy, bubble tube, wall projectors, and LED fiber optics as highly effective, followed by the Megapod and vibroacoustic furniture, as seen in Figure 4.

An interesting theme was that several respondents mentioned that the MSE not only benefited Veterans with dementia but also had a positive, calming influence on staff stress and anxiety levels. One respondent said, “I’m even sitting in front of the aquarium sometimes when I’m really agitated. I have my headphones on with the music, and I just relax with the bubbles. It’s nice” (Participant 31, Nursing Assistant).

#### Unintended negative effects of MSE.

While all 32 participants reported some positive effects from MSE, a few reported unintended negative effects. Four participants noted that Veterans with PTSD showed a heightened sensitivity to sound and lights, particularly to the fireworks display on some of the visual lighting equipment. We do not know whether these four participants were referring to the same or different Veterans. One interviewee noted, “We had one resident with PTSD and the MSE had the opposite effect and caused a flashback” (Participant 31, Recreation Therapist). Two participants also mentioned that, due to the varied progression of cognitive impairment for Veterans with dementia, some patients had a hard time understanding the MSE which led to negative or negligible impact on behavior.

The MSE could be unintentionally scary or upsetting. Some staff members noted that the various lights and sounds could inadvertently frighten Veterans with dementia. The staff said caution should be taken when first introducing the patient to the environment. One respondent noted,

Sometimes depending on what you have on could freak somebody out … some colors like red make the room feel eerie … Also smells are so strong and … can create issues if you don’t know how to use it. So, you just have to be careful. (Participant 30, Recreation Therapist)

The few participants who mentioned unintended negative effects of MSE offered possible solutions to consider. These included knowing your Veteran, understanding his or her diagnoses and preferences and reducing MSE stimuli when appropriate.

### Identifying and Overcoming Barriers to Uptake (Aim 2)

Several barriers to uptake of MSE therapy, as well as possible strategies for overcoming those barriers, were identified through coding and analysis of transcripts

#### Dedicate a room for MSE therapy.

Several staff members noted the importance of the MSE being installed within a dedicated room. Some of the participants had mobile equipment such as the MSE cart that could be moved into various areas in the CLC. A common feeling, however, was the MSE was most effective provided within a dedicated room in a permanent application. One respondent commented, “Having a dedicated room is important because you always have it up and ready to go … you need a dedicated place or it just ends up being a piece of furniture” (Participant 29, Psychologist).

#### Provide sufficient space.

Several respondents felt that the size of the room was an extremely important factor impacting the MSE’s effectiveness. A common theme was group MSE applications and the need for rooms that were large enough to accommodate Veterans and staff for MSE group therapy applications. One respondent mentioned that the rooms should be “large enough for at least 5 patients to interact in the room” (Participant 28, Nursing Assistant). Respondents felt the MSE must accommodate Veterans “from hospice and interim care;” one respondent said, “They’re [the patients] not allowed out of the bed, so we bring the bed down to the room” (Participant 1, Recreation Therapist).

#### Provide effective training.

Many participants felt the most common barrier to uptake was the need for more MSE training, mentioning that when they did receive training it was from the equipment vendor, only offered once, and focused solely on how to operate the equipment. For instance, staff emphasized that “We need more training … training is so important … and lack of training leads to maintenance problems” (Participant 6, Recreational Therapist). While another noted the need for specific training, “on how the modalities help with different behavioral issues presented by [residents]” (Participant 14, Nurse).

In addition to a lack of overall training, a few respondents cited inconsistent new staff training and cross-training as barriers to uptake. For example, one staff member commented, “We don’t really have anyone who has that as their specific duty to make use of it or train others around it, so it kind of gets forgotten” (Participant 29, Psychologist). Several respondents felt that more team members needed to be educated on the use and maintenance of the equipment and that the training should be extended to all staff who interact with Veterans with dementia across various shifts. There was a belief that staff might not be using the MSE specifically because they did not have training. This was supported by additional staff who felt that a lack of cross-training for various occupations, such as recreational therapy and nursing, also prevented uptake of the MSE.

In addition to those issues, a few respondents felt that MSE uptake was impeded by the lack of training that helped staff understand the specific purpose of MSE. This need seemed to prevent the use of the MSE at the staffing level as well as in upper administration because staff “might not feel comfortable or may not feel as if they have the adequate training to utilize the equipment” (Participant 4, Recreation Therapist), and others felt that the training should also explain the purpose as it relates to each specific piece of equipment. If the training did not explain the purpose of the MSE, staff engagement seemed to be negatively impacted. For example, one respondent referred to a barrier seen in support from executive staff by saying: “They [upper administration] don’t understand that it’s a therapeutic modality … So, there’s little hurdles and speed bumps like that” (Participant 25, Recreation Therapist). Respondents agreed overall that there is a need for training staff on how to best interact with the dementia population. Respondents also agreed that specific therapies relevant to certain behaviors are necessary, and an understanding of the importance of feedback from a mental health expert is needed.

#### Communicate lessons learned.

Many participants revealed the need for more communication throughout the VHA regarding methods of MSE application, lessons learned from other staff members, and how to identify sensory preferences of Veterans. Within these, many shared the feeling that they were operating as silos without regular contact or feedback from other CLCs using the MSE. Respondents assented that VHA staff needed a way to share successful MSE application methods and findings to help improve the uptake and application throughout the VHA. Many cited the importance of regular communication among staff so that people knew what worked and what did not work for each Veteran. One respondent noted that regular communication is incredibly important so that “the next shift doesn’t come in and do the same thing that didn’t work and can [instead] build on what does work and everybody knows” (Participant 30, Recreation Therapist).

One of the critical elements that emerged regarding the effectiveness of the MSEs is the significant impact of incorporating individual Veteran preferences into the therapy. Many respondents believed personal preferences was a vitally important element impacting MSE effectiveness. For instance, several respondents stressed the importance of matching patients’ preferences with their needs and abilities. One respondent said emphatically, “You need to know the residents who you put in there. You can’t just take somebody in there and then just push a button and say here it goes … It’s all about them” (Participant 31, Nursing Assistant).

A few participants noted gender preferences as important factors to be considered. One respondent mentioned that “female Veterans will sit in the vibroacoustic chair and braid the LED fiber optic because it looks like hair” (Participant 30, Recreation Therapist). Researchers also noted that matching the environment to the specific preferences that align with what the Veterans did throughout their career or as a hobby helped in impacting the effectiveness of the MSE.

#### Engage staff.

Although many participants reported that the MSE was well-used in their facility, a few noted that a lack of staff engagement with the MSE prevented use. A few noted that high levels of staff turnover created fluctuating staffing levels that impeded staff’s availability and willingness to engage with MSE. For example, one person remarked: “New employees are being pulled to different units, so we don’t have the same staff members who are knowledgeable and know how to use it” (Participant 6, Recreation Therapist). Another person commented that, “We have so much staff turnover, either [staff] don’t think to use it or they’re new employees and they don’t know what it is” (Participant 29, Psychologist). Several respondents felt that high turnover also led to an overall lack of time for staff remaining on the dementia wards due to the challenging behaviors of Veterans with dementia. One respondent said, “If you don’t have enough people working it’s not gonna work because there’s too much distraction [on the ward]” (Participant 31, Nursing Assistant).

Several respondents within this category felt that lack of engagement with the MSE derived from an unclear understanding of the purpose of MSE for Veterans with dementia. This seemed to negatively impact the staff’s perception of the value of the MSE. One person commented that people might therefore say, “Hey, this is not my job, I wasn’t hired to do that or that’s not in my description or duty” (Participant 6, Recreation Therapist). Another respondent stated, “It takes us a while to take stuff out and then put it back,” noting that a lack of time negatively impacted perceived value of the elements of MSE (Participant 27, Recreation Therapist). A different respondent noted that “people felt that it was time consuming … it was seen as just one more thing to do” (Participant 7, Nurse).

#### Develop a clear maintenance plan.

A few respondents felt that an unclear maintenance plan for the MSE was a significant barrier to its use. High turnover mentioned earlier seems to influence staff’s willingness to take on the perceived additional responsibility of engaging with the MSE. One participant noted, “The cleaning of the machines is difficult sometimes. Especially when you have it in a room and you take it out you have to sterilize it, wipe it down … ” (Participant 31, Nursing Assistant). Another respondent mentioned that “the electronics don’t always work. The Megapod doesn’t always work when it’s connected, or the bubble tube needs maintenance” (Participant 27, Recreation Therapist). Unclear ownership and responsibility regarding maintenance and use prevented some staff from engaging with the MSE.

#### Have equipment easily accessible.

Several participants commented that physical access to the MSE with regard to both its inconvenient proximity to Veterans who need to use the MSE and to the inability of staff to access the room itself and the limiting size was a barrier to use. For example, Veterans within hospice and palliative care in the end stages of life are unable to leave their beds, so the only way to leave their rooms was to be rolled out of their room while still in their bed. For these Veterans, travel distance and the size of the MSE room (since it would need to accommodate a hospital bed, patients, and staff) are major barriers. This relates not only to the relative proximity of the MSE to the Veterans in hospice but also to the feasibility of staff to transport the Veterans to the space by rolling large hospital beds through corridors and having the MSE large enough to accommodate such sizeable furniture.

A few participants noted that another barrier to MSE uptake involves the staff’s inability to access the room itself. To mediate the fear of equipment becoming lost or damaged, some facilities have locked the MSE rooms which doesn’t work well with staff on different shifts without the key need to access the room. One participant noted that the MSE was “locked up in a closet and no one had keys to access that closet, so that was a big problem” (Participant 29, Psychologist).

#### Empower an MSE champion.

Many respondents believed an MSE champion on staff had a positive influence on other staff members. At one site, this person actively influenced the effectiveness of the MSE by acting as an ambassador who demonstrated to other team members that she or he wants to be engaged with the MSE and sincerely wanted Veterans to do well. By having this advocate, many respondents felt that the consistency in MSE application would benefit significantly. One respondent commented, “I think what would benefit our residents is if we had an employee who was specifically trained and that was their job function” (Participant 8, Nurse).

One of the main barriers to MSE uptake involved the maintenance and management of the expensive equipment. If there was no champion, respondents felt that the MSE would decrease in effectiveness as equipment might break or need repair over time. One respondent illustrated this by stating: “Make sure that … person is identified because sometimes, like the bubble tube, they need to be cleaned and who is responsible? So, make sure that that person is identified” (Participant 4, Recreation Therapist). Staff respondents also felt that having a champion for the MSE has a positive influence on upper leadership, benefitting the MSE effectiveness and application. One respondent mentioned, “You need leadership to be on board. You need people who are higher up than you to ‘get it’ because without that there’s really no one pushing for things” (Participant 30, Recreation Therapist).

### Limitations

There are some limitations that should be considered. Rigorous VHA guidelines enforced careful restrictions on study methods and recruitment. The recruitment flyer required approval at a national level to be administered to all CLC facilities across the VHA system. The study team was required to wait for responses, which came slowly. With high staff turnover rates, many staff who had originally received the MSE had changed. This explains why only 12 sites responded to the flyer. The small size of the sample is a major limitation as the findings may not be generalizable and may contain bias relative to the respondents who participated. Also, although care was taken to mitigate bias during transcript coding, the findings are subject to biases of the coding team.

## Conclusion

Following Roger’s DOI theory, the diffusion innovation of MSE as behavioral therapy for Veterans with dementia has been successful and is nearly complete. The innovation has constructively spanned the stages of knowledge, persuasion, decision, and implementation, and this study represents the confirmation phase as an analysis of the MSE as an adopted innovation. Overall, feedback regarding the MSE at the VHA was positive as respondents felt it was effective in improving behavior, mood, and increasing engagement for Veterans with dementia. This is significant, as it suggests it may be an impactful behavioral intervention for psychosocial, physical activity, sensorial, and staff-focused interventions; four of the five nonpharmacological categories identified by Cabrera et al (2015). Input regarding staff perceptions of MSE efficacy is impactful as well because, although the built environment is known to be capable of benefiting people with dementia (Marquardt et al., 2014), little is known regarding MSE efficacy. However, the MSE was not without critique. The most commonly perceived barriers to use of the MSE were inadequate training and communication, lack of staff engagement, lack of clear maintenance, and access to the equipment. Regarding the equipment itself, the mobile MSE carts were not preferred over the dedicated room due to maintenance complexity, replacement expense, and its cumbersome size. Interestingly, many of these issues may be addressed if adequate MSE training is provided for staff and if the MSE is designed within a dedicated room that is easily accessible, with ownership clearly identified. The findings from this research indicate MSE are perceived to be effective in improving behavior for Veterans with dementia and represent an innovation that has been well-applied and has great potential for future clinical applications.

## Recommendations for Future Research

This study has added to the existing literature by being the first to analyze MSE preferences and application across the VHA, the largest healthcare system within the United States. Because MSEs were distributed throughout the VHA as part of a grant, much of the equipment was purchased from the same vendors and the applications across facilities were similar. Gathering perceptions of medical staff regarding MSE for Veterans with dementia is another significant contribution. Future research would benefit from a larger sample size with more representation of CLC sites and participants from each site or across multiple non-VHA sites to identify more generalizable findings. Building upon the findings from this study, future research can investigate which MSE elements are most effective for specific behavioral needs for Veterans with dementia by incorporating bioinformatics in more controlled studies. These findings have the potential to benefit not only people with dementia but also others with cognitive impairments including PTSD, and TBI.

## Implications for Practice

Many respondents suggested that the MSEs were most effective in dedicated rooms versus mobile cart applications, supporting the implication of the importance of designers of the built environment to incorporate MSE into the design of dementia-care facilities. This is interesting as three of the most favored elements are also within the top four sensory items with high effectiveness ratings. This indicates that these sensory elements are key items to be incorporated into dedicated MSE. Currently, these elements are pieces of equipment placed in an environment, but there is great opportunity for designers of the built environment to focus on these elements and find innovative ways to incorporate them into the space in a more impactful, cost-effective way that provides greater flexibility and stability in application.

## Figures and Tables

**Table 1.** Interview Participant Characteristics.

Characteristic	Qty
Total respondents	32
Gender	4 male, 28 female
Total sites	12 different sites
Occupations	12 registered nurses, 4 nursing assistants, 1 psychologist, 12 recreation therapists, 1 medical director medical assistant 2
Years at site	8 years average, ranging from 1 to 30 years
Site logistics	8 U.S. States, 1 U.S. Territory
